# Pooling Data on Pools: Genotoxicity of Chemicals in Indoor Swimming Pools

**DOI:** 10.1289/ehp.118-a490a

**Published:** 2010-11

**Authors:** Angela Spivey

**Affiliations:** **Angela Spivey** writes from North Carolina about science, medicine, and higher education. She has written for *EHP* since 2001 and is a member of the National Association of Science Writers

Disinfection by-products (DBPs) form in swimming pool water from reactions between disinfectants such as chlorine and organic matter such as sweat, skin cells, and urine. A new study described in a set of three articles provides the first comprehensive characterization of DBPs in an indoor pool environment and offers initial evidence of cellular-level effects of these chemicals in swimmers in an indoor chlorinated pool **[***EHP* **118(11):1523–1530; Richardson et al.;** *EHP* **118(11):1531–1537, Kogevinas et al.;** *EHP* **118(11):1538–1544, Font-Ribera et al.]**.

The authors assessed short-term changes in 49 healthy adults after they swam for 40 minutes in a public indoor chlorinated pool. They observed increases in two biomarkers of genotoxicity relative to the concentration of brominated trihalomethanes (THMs) in exhaled breath, which were used as a proxy of the swimmers’ total DBP exposures. Those biomarkers were micronuclei in blood lymphocytes (which have been associated with cancer risk in healthy subjects) and urine mutagenicity (a biomarker of exposure to genotoxic agents).

The team also took detailed measurements of THMs in air around the pool and in exhaled breath of the swimmers before and after swimming. The investigators measured several biomarkers of respiratory effects after swimming and found changes in only one—a slight increase in serum CC16, which suggests an increase in lung epithelium permeability. However, they found no evidence that DBP exposure affected lung function.

The research team identified more than 100 DBPs in the water of the chlorinated pool as well as another indoor pool disinfected with bromine. Some of these compounds had never been reported previously in swimming pool water and/or chlorinated drinking water. *In vitro* assays showed the swimming pool water was mutagenic at levels similar to that of drinking water but was more cytotoxic (could kill cells at a lower concentration) than drinking water.

The researchers acknowledge the need for further research on a variety of swimming pools under various conditions of maintenance and use as well as more complete evaluations of the uptake and potential effects of the compounds present in pool water. They also note the importance of timing in the collection of biological samples—a parameter for which there was no precedent, given the lack of previous studies of this type with swimmers. Above all, they emphasize that positive health effects of swimming can be maintained by minimizing pool levels of DBPs.

## Figures and Tables

**Figure:**
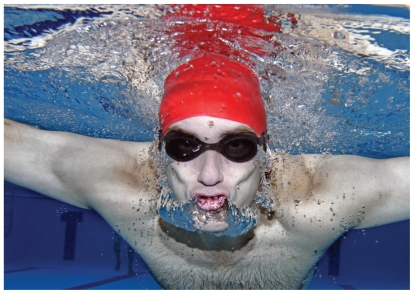
Markers of genotoxicity and mutagenicity were detected in swimmers after 40 minutes in the pool.

